# A Hybrid Multiuser Detector Based on MMSE and AFSA for TDRS System Forward Link

**DOI:** 10.1155/2014/620617

**Published:** 2014-04-22

**Authors:** Zhendong Yin, Xu Jiang, Zhilu Wu, Xiaohui Liu

**Affiliations:** School of Electronics and Information Engineering, Harbin Institute of Technology, Harbin 150001, China

## Abstract

This study mainly focuses on multiuser detection in tracking and data relay satellite (TDRS) system forward link. Minimum mean square error (MMSE) is a low complexity multiuser detection method, but MMSE detector cannot achieve satisfactory bit error ratio and near-far resistance, whereas artificial fish swarm algorithm (AFSA) is expert in optimization and it can realize the global convergence efficiently. Therefore, a hybrid multiuser detector based on MMSE and AFSA (MMSE-AFSA) is proposed in this paper. The result of MMSE and its modified formations are used as the initial values of artificial fishes to accelerate the speed of global convergence and reduce the iteration times for AFSA. The simulation results show that the bit error ratio and near-far resistance performances of the proposed detector are much better, compared with MF, DEC, and MMSE, and are quite close to OMD. Furthermore, the proposed MMSE-AFSA detector also has a large system capacity.

## 1. Introduction

In 1963, due to the limited coverage of low-altitude orbiting spacecraft by a practical number of ground stations, F. O. Vonbun conceived the idea of tracking and data relay satellites (TDRS). Decades of space technology development now offer a practical extension from present ground-to-ground and air-to-ground communication via satellites to new applications [1].

Tracking and data relay satellite system (TDRSS) can provide services of data relaying, continuous tracking, and telemetry tracking and command (TT&C) for communications between spacecraft such as low earth orbit (LEO), middle earth orbit (MEO), and ground stations, which constitute important part of global space-based integrated information networks [2]. TDRSS provides S-band services through the S-band multiple access (SMA) phased array [3]. Actually the multiple access interference (MAI) is a serious limiting condition for improving the performance and the user capacity of this MA system, particularly when the number of users in this system is large.

Multiuser detection is a useful method to eliminate the bad effect of MAI. The best performance is acquired by OMD provided by Verdu in 1986, which is based on the maximum likelihood function [4]. However, this method tends to be quite complex. Consequently, multiuser detectors based on compressive sensing [5], Tikhonov regularization [6], ant colony optimization [7], adaptive LMS, and GA [8] have been devoted to the development of lower-complexity techniques that can achieve some of the benefits of the optimal procedures. However, the tradeoff problem between computational complexity and BER performance still exists.

Swarm intelligence (SI) is an innovative artificial intelligence technique for optimization [9]. The underlying perception in most of the biological case studies of SI has been that the individual animal is cognitively relatively simple and restricted in what it can achieve, whereas the group collectively is capable of astonishing feats [10]. As ones of the latest methods in the field of signal processing [11] (especially for combinatorial optimization problems [12]), several detectors based on swarm intelligence, such as ant colony optimization [13], particle swarm optimization [14, 15], and improved particle swarm optimization [16], have been considered. The artificial fish swarm algorithm (AFSA) reflects many excellent properties in applications such as insensitivity to initial values, strong robustness and much flexibility in practice, optimization precision, rapidness to search the global optimum, tolerance of parameter setting, and searching adaptation [17]. It is applied in various optimization applications such as solving III-conditioned linear systems of equations [18] and reactive power optimization for power system [19]. And several improved AFSAs have been proposed [20, 21]. In [20], an artificial fish swarm algorithm based on chaos search is proposed, which can not only overcome the disadvantage of easily getting into the local optimum in the later evolution period but also keep the rapidity of the previous period. In [21], two artificial fish swarm algorithms based on fuzzy system are proposed; the overall results show that proposed algorithms can surprisingly be effective.

In this paper, a multiuser detector in TDRS system forward link is employed. In order to accelerate the speed of global convergence and reduce the number of iterations for AFSA, the result of MMSE and its modified formations are used as the initial values of artificial fishes. Experimental results demonstrate that the BER and near-far resistance performances of the proposed MMSE-AFSA detector are better, compared with matched filter (MF), decorrelating detector (DEC), and MMSE, and are quite close to OMD.

This paper is organized as follows.  Section 2 introduces the system model of multiuser detector in TDRS systems and several existing detectors. In Section 3, basic principle of AFSA and the proposed MMSE-AFSA detector are illustrated. Then in Section 4, experiments that compare with the performances of MF, DEC, MMSE, MMSE-AFSA, and OMD are analyzed. The paper is concluded in Section 5.

## 2. System Model and Several Existing Methods

### 2.1. System Model

Consider a TDRS system with S-band code division multiple access (CDMA). Assume there are *K* simultaneously active users. Over additive white Gaussian noise (AWGN) channel, the equivalent low-pass received waveform can be expressed as

(1) $$\begin{array}{c} y\left(t\right)=\overset{K}{\underset{k=1}{\sum }}\sqrt{E_{k}}s_{k}\left(t\right)b_{k}+n\left(t\right)\;0\leq t\leq T, \end{array}$$

where *E*_*k*_, *s*_*k*_(*t*), and *b*_*k*_ ∈ {−1, 1} represent energy per bit, unit-energy signature waveform, and bit value of the* k*th user, respectively, *n*(*t*) is the noise, and *T* is the bit interval.

The output of the matched filter of user *k* sampled at *T* is achieved by the following equation:

(2) $$\begin{array}{c} y_{k}=\overset{T}{\underset{0}{\int }}y\left(t\right)s_{k}\left(t\right)\text{d}t=\sqrt{E_{k}}b_{k}+\overset{K}{\underset{\begin{array}{c} i=1\\ i\neq k \end{array}}{\sum }}\sqrt{E_{i}}\rho_{ik}b_{i}+n_{k}, \end{array}$$

where the noise at the output of the* k*th matched filter is *n*_*k*_ = ∫_0_^*T*^ *s*_*k*_(*t*)*n*(*t*)d*t*, and the cross correlation of the signature waveforms of users *i* and *k* is *ρ*_*ik*_ = ∫_0_^*T*^ *s*_*i*_(*t*)*s*_*k*_(*t*)d*t*.

The matched filter outputs can be expressed in vector form as follows:

(3) $$\begin{array}{c} y={\left[y_{1}y_{2}\ldots y_{K}\right]}^{T}=RAb+n, \end{array}$$

where **R** is the normalized cross correlation matrix of the signature waveforms, **R**_*ij*_ = *ρ*_*ij*_, $A=\mathrm{diag}{\left[\sqrt{E_{1}}\sqrt{E_{2}}\cdots \sqrt{E_{K}}\right]}_{K\times K}$, and **n** is the zero-mean AWGN noise vector.

The symbol decisions of matched filter are given by

(4) $$\begin{array}{c} \hat{b}=\mathrm{sgn}\left(y\right). \end{array}$$

### 2.2. Decorrelating Detector

Numerous suboptimal approaches to multiuser detection have been proposed to trade off performance and complexity. A widely studied linear solution is decorrelating detector. In this category, the decorrelator completely eliminates the MAI by orthogonalizing the users. The transformation **R** ^−1^ is applied to the output of matched filters; the symbol decisions are given by

(5) $$\begin{array}{c} {\hat{b}}_{\text{DEC}}=\mathrm{sgn}\left(R^{-1}y\right)=\mathrm{sgn}\left(Ab+R^{-1}n\right). \end{array}$$

It can be immediately inferred that each component of the decision vector **y**_dec_ is interference-free. On the other hand, the background noise can be enhanced by the transformation **R** ^−1^.

### 2.3. Minimum Mean Square Error Detector

Another important linear detector is minimum mean square error detector. The aim of MMSE detector is to choose the *K* × *K* matrix **M** that minimizes

(6) $$\begin{array}{c} \Omega \left(M\right)=\min E\left\{{||b-My||}^{2}\right\}. \end{array}$$

It can be easily seen that **M** = **A** ^−1^[**R**+*σ* ^2^ **A** ^−2^]^−1^ is the solution to (6). The symbol decisions are

(7) $$\begin{array}{c} {\hat{b}}_{\text{MMSE}}=\mathrm{sgn}\left(My\right). \end{array}$$

It balances the desire to completely eliminate the MAI with the desire to avoid the background noise enhancement.

### 2.4. Optimal Multiuser Detector

On the basis of matched filter, optimal detector takes advantage of the maximum likelihood sequence detection algorithm to improve the performance of multiuser detector. The likelihood function of *y* given *b* is given by

(8) $$\begin{array}{c} p\left(y\;|\;b\right)=\exp\left(\frac{-\left(1/2\right){\left(y-RAb\right)}^{T}{\left(\sigma^{2}R\right)}^{-1}\left(y-RAb\right)}{{\left(2\pi \right)}^{K/2}\sigma {\left|R\right|}^{1/2}}\right), \end{array}$$

where |**R**| denotes the determinant of **R**. The maximum likelihood symbol decisions are determined as

(9) $$\begin{array}{c} {\hat{b}}_{\text{OMD}}=\arg\;\underset{b}{\max}\left\{2b^{T}Ay-b^{T}ARAb\right\}. \end{array}$$

The above maximization problem is a combinatorial optimization problem which is known to be NP-hard: its computational complexity increases exponentially with the number of users in TDRS system. This *O*(2^*K*^) implementation complexity required by OMD makes it impractical for real system. OMD represents, however, a basis for comparison for other suboptimal detectors.

## 3. MMSE-AFSA Detector

### 3.1. Basic Principles of AFSA

Artificial fish swarm algorithm is a new bionic optimization algorithm based on the study of fish swarm's intelligence and behaviors in nature. There are mainly three types of fish behaviors: preying behavior, swarming behavior, and following behavior. The general AFSA is introduced below.

#### 3.1.1. Several Definitions for AFSA

In the AFSA, suppose there are *n* artificial fishes. The state of each artificial fish can be expressed as a *K*-dimensional vector **X** = (*x*_1_,*x*_2_,…,*x*_*K*_)^*T*^. The objective function **Y** = *f*(**X**) denotes the food concentration level of this state. The distance between states **X**_*i*_ and **X**_*j*_ is defined as

(10) dij=||Xi−Xj||=(xi1−xj1)2+(xi2−xj2)2+⋯+(xiK−xjK)2.

Besides,* Visual *denotes the local visual (or searching) distance of artificial fishes;  *δ*  is the factor of crowdedness that affects the number of artificial fishes in the local space; step is the movement size of artificial fishes;* try_number* is the random searching times in preying behavior.

#### 3.1.2. Behaviors of AFSA

*Preying Behavior*. Suppose that the current state of an artificial fish is **X**_*i*_. **X**_*j*_ is a random state chosen in its visual field. In the maximum problem, if *f*(**X**_*j*_) > *f*(**X**_*i*_), this artificial fish will move from state **X**_*i*_ to **X**_*j*_ as

(11) $$\begin{array}{c} X_{i\text{next}}=X_{i}+\text{rand}\left(0,1\right)\times \text{step}\times \frac{X_{j}-X_{i}}{||X_{j}-X_{i}||}. \end{array}$$

Otherwise, choose a new state **X**_*j*_ randomly again and judge whether it satisfies the movement condition (*f*(**X**_*j*_) > *f*(**X**_*i*_)). If there is no such **X**_*j*_ that can satisfy this condition after trying* try_number* times, this artificial fish will move one step randomly at last

(12) $$\begin{array}{c} X_{i\text{next}}=X_{i}+\text{rand}\left(0,1\right)\times \text{step}. \end{array}$$

*Swarming Behavior*. The current state of an artificial fish is **X**_*i*_, and *n*_*f*_ is the number of companions within its visual range. Thus, the central state of these artificial fishes is given by

(13) $$\begin{array}{c} X_{c}=\overset{n_{f}}{\underset{j=1}{\sum }}\frac{X_{j}}{n_{f}}. \end{array}$$

If *f*(**X**_*c*_)/*n*_*f*_ > *δf*(**X**_*i*_), which means the food concentration of **X**_*c*_ is sufficient and this area is not too crowded, then this artificial will move to the central state **X**_*c*_ as

(14) $$\begin{array}{c} X_{i\text{next}}=X_{i}+\text{rand}\left(0,1\right)\times \text{step}\times \frac{X_{c}-X_{i}}{||X_{c}-X_{i}||}. \end{array}$$

Otherwise, preying behavior will be executed.

*Following Behavior*. Within the visual range of **X**_*i*_, **X**_max⁡_ denotes the state whose food concentration *f*(**X**_max⁡_) is maximum. If *f*(**X**_max⁡_)/*n*_*f*_ > *δf*(**X**_*i*_) and *f*(**X**_max⁡_) > *f*(**X**_*i*_), this artificial fish will move to state **X**_max⁡_ as follows:

(15) $$\begin{array}{c} X_{i\text{next}}=X_{i}+\text{rand}\left(0,1\right)\times \text{step}\times \frac{X_{\max}-X_{i}}{||X_{\max}-X_{i}||}. \end{array}$$

Otherwise, preying behavior will be executed.

#### 3.1.3. Bulletin Board

A bulletin board is established to record the optimal state and the optimal value of these artificial fishes. Each artificial fish will compare its current state to the state on the bulletin board. If its food concentration is better, update the bulletin board with the better state.

#### 3.1.4. Behavior Selection

**Figure 1:**
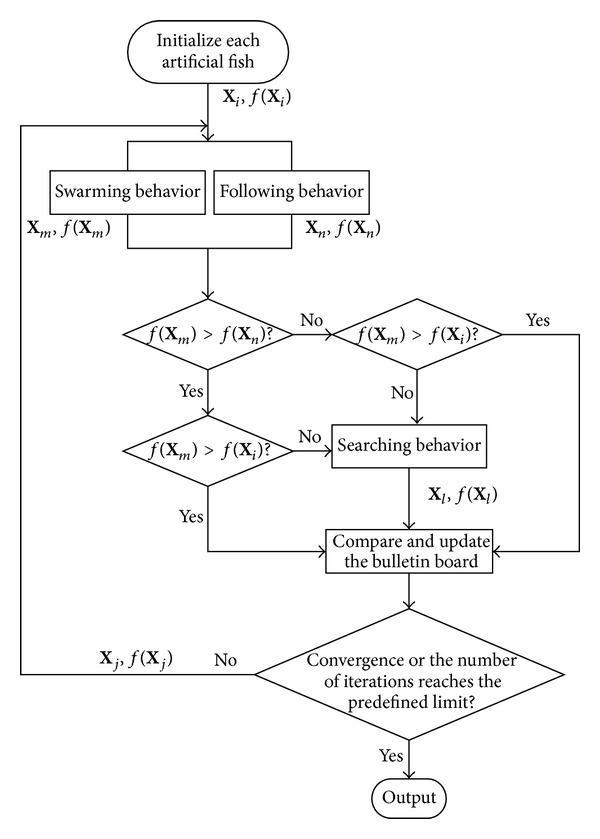
The behavior of each artificial fish in AFSA.

Evaluate the current environment of artificial fishes according to the problem to be solved, and then select a behavior. In the maximum problem, simulate swarming behavior and following behavior of each artificial fish and compare the food concentration of two behaviors, and the better behavior will be implemented. If none of them can improve the former state of the certain artificial fish, preying behavior will be executed. The behavior of each artificial fish in AFSA is shown in Figure 1.

### 3.2. The Discretization of AFSA

The process of OMD is similar to that of a function's optimization. Whereas AFSA is expert in optimization and it can realize the global convergence efficiently, the optimization function for OMD is shown in (9), which is a discrete optimization function. Therefore, the model of AFSA should be discretized. AFSA applied to multiuser detection problem with some additional explications in the discrete Euclidean solution space **E** ^*K*^ are expressed as follows.

(1) In the Euclidean solution space **E** ^*K*^, the state of each fish is encoded by +1 or −1. If there are *K* active users in a TDRS system, the state is a *K*-dimensional vector, like **X** = (*x*_1_,*x*_2_,…,*x*_*K*_)^*T*^, where *x*_*i*_ ∈ {+1, −1}, *i* = 1, 2, …, *K*.
(2) The initial value of each artificial fish is selected randomly in the discrete solution space with 2^*K*^ likely solutions.
(3) In this case, the operator XOR is used to calculate distance between states of two artificial fishes. For instance, the state of an artificial fish **X**_*i*_ = (+1, + 1, −1, +1, −1), the state of another artificial fish **X**_*j*_ = (+1, −1, +1, −1, +1), then the distance between the two artificial fishes *d*_*ij*_ = **X**_*i*_ XOR **X**_*j*_ = 4.
(4) The central state of a certain artificial fish is given by
  (16) $$\begin{array}{c} X_{c}=\mathrm{sgn}\left(X_{1}+X_{2}+\cdots +X_{n}\right), \end{array}$$
  where *n* is the number of artificial fishes.
(5) The fitness function for AFSA is the criterion of OMD given by
  (17) $$\begin{array}{c} f\left(X\right)=2X^{T}Ay-X^{T}ARAX, \end{array}$$
  where **X** is the state of a certain artificial fish.
(6) Equations (11), (14), and (15) are, respectively, modified as follows:
  (18) Xinext =Xj,Xinext =Xc,Xinext =Xmax⁡.

### 3.3. The Procedure of the Proposed MMSE-AFSA Detector

Since AFSA is a random searching swarm intelligence algorithm, the initial values have a great effect on its convergence speed. This suggests that, in order to decrease the number of iterations, the initial states of these artificial fishes should be selected with the a priori knowledge rather than selected randomly. Therefore, a novel MMSE-AFSA detector is proposed here. The result of MMSE and its modified formations are used as the initial values of artificial fishes. The initialization of artificial fishes is described below.

**Step 1**

Execute MMSE detector to get a suboptimal solution. Assign the result **b**_1_ = (*b*_11_,*b*_12_,…,*b*_1*K*_)^*T*^ as the initial state of the first artificial fish, where *b*_1*i*_ ∈ {+1, −1} and *i* = 1, 2,…, *K*.

**Step 2**

Then, randomly change an element *b*_1*i*_ of **b**_1_; that is, let *b*_2*i*_ = −*b*_1*i*_. And assign the new state **b**_2_ which is modified from **b**_1_ to another artificial fish.

**Step 3**

Repeat Step 2 and initialize the rest of the artificial fishes in the same way.

**Figure 2:**
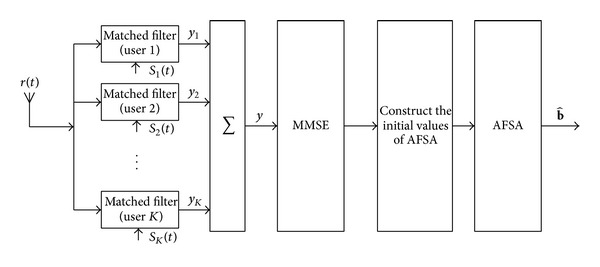
Diagram of MMSE-AFSA detector.

After initialization, run AFSA to get the optimal solution of multiuser detection. As described above, the overall structure of MMSE-AFSA detector is shown in Figure 2.

### 3.4. Convergence Analysis of the Proposed Algorithm

After each iteration in this algorithm, preying behavior obviously provides a better solution than the previous solution; swarming behavior improves the state of each artificial fish in their own visual range; artificial fishes move towards the optimal state within their visual range after following behavior. The behavior selection described in Section 3.1.4 chooses the best behavior after each alteration. All these processes are beneficial to the convergence of the proposed algorithm.

Besides, appropriate parameters have great influence on the convergence of the algorithm. A smaller* try_number* helps artificial fishes to avoid local optimum and move towards global optimal solution. Artificial fishes are easier to find global optimal solution with a bigger* Visual*, whereas smaller* try_number* and bigger* Visual* usually mean higher computational complexity.

The number of artificial fishes also affects the performance of the algorithm. With more artificial fishes, the algorithm is easier to converge and achieve global optimum. However, the price is higher computational complexity.

So appropriate parameters and proper number of artificial fishes are beneficial to the convergence of the proposed algorithm.

## 4. Simulations and Discussions

In this Section, Monte Carlo simulations are utilized to verify the proposed MMSE-AFSA detector. And the performances of MF, DEC, MMSE, OMD, and MMSE-AFSA are compared over AWGN channel. Most of the parameters used for these simulations are summarized in Table 1.

### 4.1. The BER Performance versus *E*_*b*_/*N*_0_

**Figure 3:**
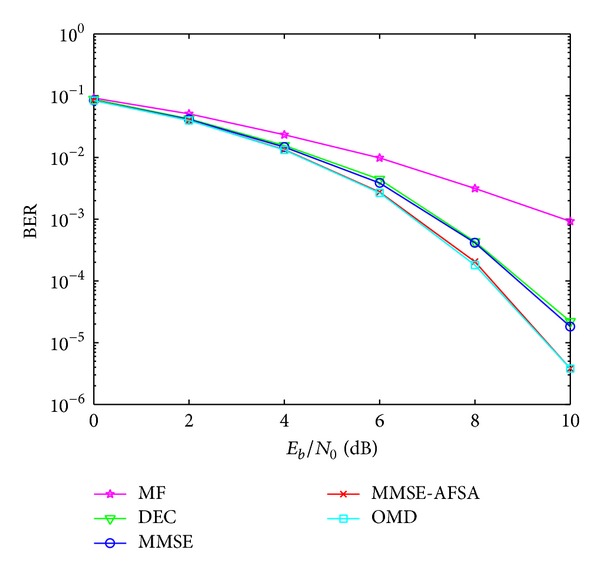
The BER performance versus *E*_*b*_/*N*_0_.

The BER performance versus *E*_*b*_/*N*_0_ with perfect power control over AWGN channel is shown in Figure 3. There are 10 users in the TDRS system and *E*_*b*_/*N*_0_ ranges from 0 to 10.

It can be easily seen from Figure 3 that the BER performance versus *E*_*b*_/*N*_0_ of MMSE-AFSA is superior compared with MF, DEC, and MMSE. In addition, it even coincides with OMD. MMSE is a suboptimal method of multiuser detection, and AFSA can efficiently find the optimal solution with the result of MMSE and its modified formations as initial states of artificial fishes. Rather than random initial values, MMSE and its modified formations are approximations of the optimal solution. That is the reason why the BER performance of MMSE-AFSA is quite close to OMD and why only 5 iterations are needed in MMSE-AFSA.

### 4.2. The BER Performance versus Number of Users *K*

The BER performance curves of these detectors with different number of active users are explored here. In this experiment, *E*_*b*_/*N*_0_ is set to 5 for all the detectors.

**Figure 4:**
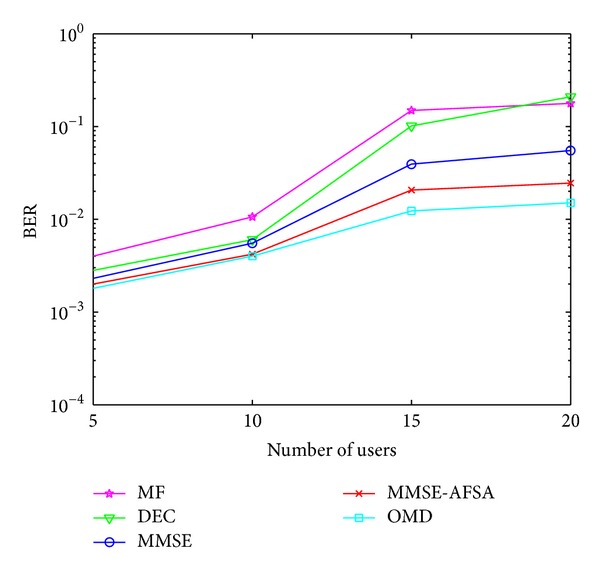
The BER performance versus user number *K*.

Figure 4 shows the simulation results. As an overall trend, BER of all the detectors increases when there are more active users in the system. OMD shows the best BER performance versus the number of active users among all these detectors. The performance of MMSE-AFSA is also better than MF, DEC, and MMSE. In this experiment, as the number of users increases, the solution space expands, while the parameters such as* Visual* and* try_number* of AFSA remain unchanged. Thus, there exists a gap between MMSE-AFSA and OMD.

### 4.3. The Near-Far Resistance of MMSE-AFSA

**Figure 5:**
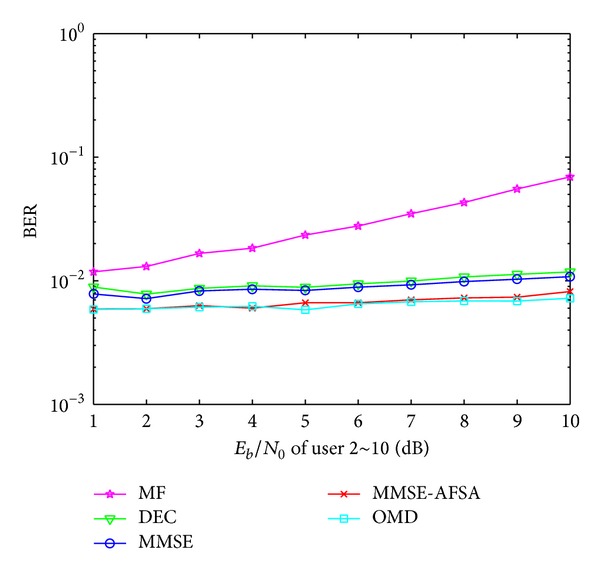
The near-far resistance of different detectors.

In this experiment, the BER performance of these detectors with imperfect power control is employed. The user number is set to 10 and *E*_*b*_/*N*_0_ of the first user is 5. While *E*_*b*_/*N*_0_ of the remaining users changes from 1 to 10 simultaneously. Simulation results, compared with MF, DEC, MMSE, and OMD, are shown in Figure 5.

As is revealed in Figure 5, OMD shows the best near-far resistance, while MF shows the worst. The near-far resistance performance of MMSE-AFSA is better than MF, DEC, and MMSE. MMSE-AFSA takes advantages from the suboptimal result of MMSE and AFSA is expert in optimization and it can realize the global convergence efficiently.

### 4.4. Different Initial States of Artificial Fishes

**Figure 6:**
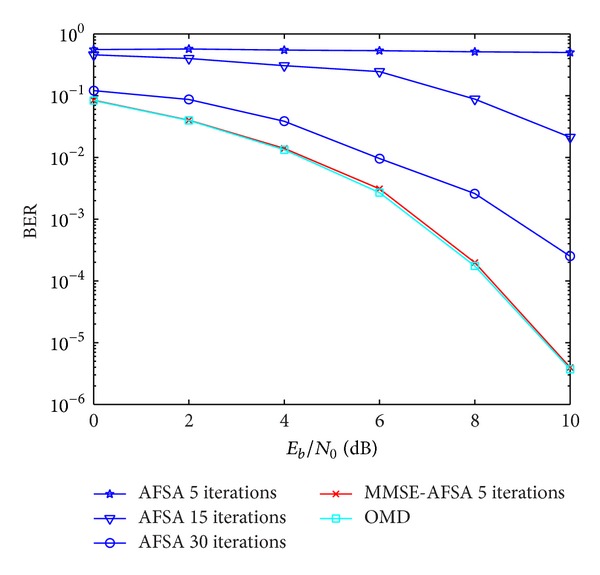
Convergence rate of AFSA detector and MMSE-AFSA detector.

As an iterative optimization scheme, the convergence rate reflects the computational complexity. Different initial values have great influence on iteration times. In this experiment, an AFSA detector whose initial values are generated randomly and the MMSE-AFSA detector whose initial values are the result of MMSE and its modified formations are discussed. The BER performance of different iteration times is shown in Figure 6, respectively. The number of users is 10.

From Figure 6, we can see that, even after only 5 times of iterative in MMSE-AFSA detector, the BER performance is quite close to OMD. However, the BER performance of AFSA detector with random initial states is worse than MMSE-AFSA despite the number of iterations being 30. It is because that there are only 3 artificial fishes, and they cannot reach global optimum easily with randomly selected initial values.

### 4.5. Computational Complexity Analysis

In order to measure the computational complexity of these detectors, relative execution time is used in this experiment. Let the execution time of MF be equal to 1; the relative execution time of DEC, MMSE, MMSE-AFSA, and OMD is shown in Table 2, respectively (suppose there are 10 active users and *E*_*b*_/*N*_0_ = 5; other simulation parameters are the same as shown in Table 1).

It can be seen from Table 2 that the computational complexity of MMSE-AFSA increases slightly compared with MMSE and is much lower than that of OMD, because, in a *K*-user TDRS system forward link, the number of iterations of OMD is 2^*K*^ for OMD is known to be NP-hard. DEC and MMSE are linear detectors so that they have a low computational complexity. The computational complexity of MMSE-AFSA contains two parts. The first part is the computational complexity of MMSE; another part is the complexity of AFSA. From Section 4.4, we can see that only 3 artificial fishes and 5 iterations are needed for MMSE-AFSA to coincide performance of OMD.

## 5. Conclusion

In this paper, a hybrid multiuser detector based on MMSE and AFSA in TDRS system forward link is explored. In order to apply AFSA in multiuser detection, the discretization of AFSA is employed. Then the result of MMSE and its modified formations are used as the initial values of discrete artificial fishes. Simulation results demonstrate that the BER performance, user capacity, near-far resistance, and computational complexity of MMSE-AFSA are superior, compared with MF, DEC, and MMSE, and are quite close to OMD. Besides, the convergence rate of the novel MMSE-AFSA detector is much quicker than AFSA detector.

## Figures and Tables

**Table 1:** Simulation parameters.

Parameter	Value
Communication link	Forward link
Multiple access	CDMA
Modulation	QPSK
Spreading codes	Gold sequences
Length of spreading codes	1023
Communication channel	AWGN
Number of tested information bits	1,000,000
Number of artificial fishes	3
*Visual *	4
*try_number *	3
Number of iterations	5

**Table 2:** Relative computational complexity of different detectors.

MF	DEC	MMSE	MMSE-AFSA	OMD
1	1.42	1.67	2.13	76.9
